# Dream Recall Frequencies and Dream Content in Wilson's Disease with and without REM Sleep Behaviour Disorder: A Neurooneirologic Study

**DOI:** 10.1155/2016/2983205

**Published:** 2016-03-07

**Authors:** Gotthard G. Tribl, Mateus C. Trindade, Michael Schredl, Joana Pires, Iris Reinhard, Thais Bittencourt, Geraldo Lorenzi-Filho, Rosana Cardoso Alves, Daniel Ciampi de Andrade, Erich T. Fonoff, Edson Bor-Seng-Shu, Alexandre A. Machado, Manoel J. Teixeira, Egberto R. Barbosa

**Affiliations:** ^1^Division of Neurology and Neurosurgery, Hospital das Clinicas, University of Sao Paulo School of Medicine, Avenida Dr. Eneas de Carvalho Aguiar, 255, 5° Andar, Sala 5084, 05403-900 Pinheiros, SP, Brazil; ^2^Sleep Laboratory, Pulmonary Division, InCor, University of Sao Paulo School of Medicine, Avenida Dr. Eneas de Carvalho Aguiar 44, 05403-000 Cerqueira Cesar, SP, Brazil; ^3^Sleep Laboratory, Central Institute of Mental Health, Medical Faculty Mannheim/Heidelberg University, J 5, 68159 Mannheim, Germany; ^4^EEG and Sleep Laboratory, Department of Neurosciences, Santa Maria University Hospital, Avenida Prof. Egas Moniz, 1649-035 Lisbon, Portugal; ^5^Electroencephalography and Clinical Neurophysiology Center (CENC), Rua Conde das Antas n°5, 1070-068 Lisbon, Portugal; ^6^Department of Biostatistics, Central Institute of Mental Health, Medical Faculty Mannheim/Heidelberg University, J 5, 68159 Mannheim, Germany

## Abstract

*Objective*. Violent dream content and its acting out during rapid eye movement sleep are considered distinctive for rapid eye movement sleep behaviour disorder (RBD). This study reports first quantitative data on dreaming in a cohort of patients with treated Wilson's disease (WD) and in patients with WD with RBD.* Methods*. Retrospective questionnaires on different dimensions of dreaming and a prospective two-week home dream diary with self-rating of emotions and blinded, categorical rating of content by an external judge.* Results*. WD patients showed a significantly lower dream word count and very few other differences in dream characteristics compared to age- and sex-matched healthy controls. Compared to WD patients without RBD, patients with WD and RBD reported significantly higher nightmare frequencies and more dreams with violent or aggressive content retrospectively; their prospectively collected dream reports contained significantly more negative emotions and aggression.* Conclusions*. The reduction in dream length might reflect specific cognitive deficits in WD. The lack of differences regarding dream content might be explained by the established successful WD treatment. RBD in WD had a strong impact on dreaming. In accordance with the current definition of RBD, violent, aggressive dream content seems to be a characteristic of RBD also in WD.

## 1. Introduction

Dream content reflects waking-life experiences, current concerns, and waking-life symptoms [1]. Since the times of the healing temples of the god Asclepius in ancient Greece, dream reports were used in medical diagnostics [2]. In modern medicine only the diagnosis of the REM sleep behaviour disorder (RBD) is based on the clinical history of dream enactment behaviour, presumed to occur during REM sleep, in addition to the polysomnographic documentation of REM sleep without atonia (RWA) [3]. As a critical feature of dreaming in RBD, in both its idiopathic and synucleinopathy-associated forms, higher amounts of aggressive dream contents have been reported [4–6]. However, it is not clear whether dreams of RBD patients contain in general more movement or aggression or only those dreams, which are acted out [7–9].

The main manifestations of Wilson's disease (WD) are hepatic dysfunction and a broad spectrum of movement disorders with parkinsonian, dystonic, ataxic, and choreatic characteristics. Cognitive limitations, especially in the executive domain [10], as well as depression are frequent in WD [11] and might contribute to altered dreaming. On the other hand, in some movement disorder conditions, physical motor disability has been shown to affect dream content very little [12, 13], as these patients generally dream of themselves without limitations of their movement abilities. In addition, RBD has been recently described also in WD [14]. Polysomnography parameters and sleep quality have been shown to be significantly worse in WD, both with and without RBD, as compared to healthy controls [15]. Diseases, which structurally affect the brain like stroke, traumatic brain injury, neoplasm, Parkinson's disease, Alzheimer's disease, fatal familial insomnia, and sporadic Creutzfeldt-Jakob disease, have been shown to have impact on dreaming [16, 17]. In a study in WD, a single question on “presence of vivid dreams” was more often answered positively in patients than in controls, but the difference was not statistically significant [18]. A questionnaire study on sleep in WD found that nightmares were reported less often by WD patients than by randomly selected controls and less often by male than by female WD patients [19].

As no data on dream frequencies and content in WD have been published, this study was performed in a prospective design to characterize dreaming in WD and in RBD in WD. Therefore, we investigated dream and nightmare recall frequencies and dream content in WD patients versus healthy, age- and sex-matched controls and in WD patients with RBD (WD + RBD) versus WD patients without RBD (WD − RBD).

We hypothesized the following. (1) In WD, dream recall frequency (DRF) and dream word count are reduced and dream content is more negative as compared to healthy controls due to the cognitive impairment in patients with WD. (2) Dreams of patients with WD show increased concerns about motor disability as compared to healthy controls as this is a major impairment in WD. (3) Patients with WD + RBD have more negative and aggressive dream content than patients with WD − RBD.

## 2. Methods

### 2.1. Patients and Healthy Controls

Patients were recruited from the movement disorders outpatient clinic at the Sao Paulo University Hospital, Sao Paulo, Brazil. Age- and sex-matched controls were recruited from medical students and administrative hospital staff. The local ethics committee of the University of Sao Paulo approved the study; written informed consent was obtained from all individual participants. All participants underwent extended and structured face-to-face interviews and clinical examinations by two board certified neurologists.

In a prior publication, we have reported on sleep characteristics of a cohort of 41 patients with WD, including five patients with WD and RBD [15]. Diagnostic procedures and the video-polysomnography documented sleep disorder have been published before [15]. The present study reports on the dream characteristics of this cohort. Due to their WD, three patients, one of them a male patient with RBD, were incapable of communicating on their dream characteristics and had to be excluded from the present study. Thirty-eight WD patients of all severity of illness levels but with preserved capacity to communicate were included; Unified Wilson's Disease Rating Scale (UWDRS) was 68.82 ± 41.12 (range 18–186). Present WD manifestation was clearly predominantly affecting the brain (UWDRS neurological subscore 50.76 ± 34.22) with almost no hepatic symptoms (UWDRS 5.87 ± 3.02). WD was diagnosed biochemically and/or genetically, according to established guidelines [20]. RBD was diagnosed according to ICSD-3 criteria (International classification of sleep disorders, 3rd ed.) [3]. RWA was determined in 32 WD patients by quantification of submental and bilateral flexor digitorum superficialis electromyogram in a whole night video-polysomnography [21]; 4 out of 38 (10%) WD patients fulfilled criteria for RBD. At examination, 12 WD patients suffered symptoms of psychiatric diseases: depression (*n* = 11), personality disorder (*n* = 3), anxiety disorder (*n* = 2), and attention deficit disorder (*n* = 1). 11 WD patients took a psychotropic medication: antidepressants (*n* = 9), benzodiazepines (*n* = 5; i.e., clonazepam *n* = 3 [range 1–8 mg/d], lorazepam *n* = 1, and clobazam *n* = 1), and neuroleptics (*n* = 2). Apart from one patient on amitriptyline 75 mg/d due to depression and another one on clonazepam 0.5 mg/d, WD + RBD patients did not suffer from psychiatric diseases and were free of a psychotropic medication.

41 age- and sex-matched, healthy controls were free of all of the following: neurological and hepatic diseases, treatment requiring sleep disorders, posttraumatic stress disorder, abuse of alcohol, and illegal drugs for the lifetime; any other psychiatric disorders and intake of antidepressant, benzodiazepine, and neuroleptic medication during the last year. 18 controls underwent the same video-polysomnography protocol as the patients.

### 2.2. Cognitive, Affective, and Sleep Questionnaires

Addenbrooke's Cognitive Examination Revised [22] is a brief test sensitive to the early stages of dementia, such as mild cognitive impairment. It examines five cognitive domains: attention/orientation (18 points), memory (26), verbal fluency (14), language (26), and visuospatial abilities (16), with a maximum total score of 100. Addenbrooke's Cognitive Examination Revised has a very good reliability with an alpha coefficient = 0.8. Cut-offs were defined at 88 (sensitivity = 0.94, specificity = 0.89) and at 82 (sensitivity = 0.84, specificity = 1.0). The likelihood of dementia was 100 : 1 at a cut-off of 82. To control for symptoms of depression and for sleep quality, the Beck Depression Inventory [23] and the Pittsburgh Sleep Quality Index [24] were implemented.

### 2.3. Dream Questionnaires

#### 2.3.1. Dream and Nightmare Recall Frequencies

Overall DRF during the last months was examined by a seven-point rating scale (0 = never, 1 = less than once a month, 2 = about once a month, 3 = twice or three times a month, 4 = about once a week, 5 = several times a week, and 6 = almost every morning) [25]. This scale showed a retest reliability for an average interval of 55 days of *r* = 0.85 (*n* = 198; [26]). Using the class means, the scale was recoded to obtain units of mornings with dream recall per week (0 → 0, 1 → 0.125, 2 → 0.25, 3 → 0.625, 4 → 1.0, 5 → 3.5, and 6 → 6.5). To measure the nightmare frequency, an eight-point rating scale [27] was presented (“how often did you experience nightmares during the last months?” 0 = never, 1 = less than once a year, 2 = about once a year, 3 = about 2 to 4 times a year, 4 = about once a month, 5 = about 2 to 3 times a month, 6 = about once a week, and 7 = several times a week). The term “nightmares” was not further defined in the instructions. To obtain units in frequency per month, class means were used to recode the scales (0 → 0, 1 → 0.042, 2 → 0.083, 3 → 0.25, 4 → 1.0, 5 → 2.5, 6 → 4.0, and 7 → 12.0). The class means were deduced from the categories, not by mathematical formula.

#### 2.3.2. Relationship between WD and Dreaming

Patients were asked to answer the following question in written form: “did you ever observe any relationship between your Wilson's disease and your dreams?” We strictly avoided inducing any possible answer beyond the free response of the participant.

#### 2.3.3. RBD Questionnaire-Hong Kong (RBDQ-HK)

The 13-item self-reported RBDQ-HK has been developed to diagnose and to monitor RBD [28]. The first five items ask dream and nightmare features. The lifetime items can be answered in three categories: “do not know,” “no,” or “yes”, coded as 0, 0, or 1. Recent 1-year frequency items are rated on a five-point scale (0 = none, 1 = yes/once or few times per year, 2 = once or few times per month, 3 = 1-2 times per week, and 4 = 3 times or above per week). The psychometric properties of the RBDQ-HK were sensitivity 82.2%, specificity 86.9%, positive predictive value 86.3%, negative predictive value 83.0%, and high internal consistency (Cronbach's alpha coefficients 0.90 for the overall scale and 0.86 for the dreams-related items) and test-retest reliability (test-retest coefficients 0.89 for lifetime and 0.80 for recent 1-year frequency scales).

### 2.4. Last Remembered Dream

The participants were asked to write down the last dream they could remember with as many details and as completely as possible [29]. They were to describe the situation in the dream, whether known or unknown, the persons, their sex, age, and relationship, and if there were animals. They were also asked to describe their feelings during the dream and if they were positive or negative.

### 2.5. Dream Diary

Subjects were required to keep a dream diary during a two-week period [30]. They were asked to write down, in the morning, immediately upon awakening and as completely as possible, the dreams they had experienced during the past night. Participants were also asked to mark if they did not remember any dream or if they remembered to have dreamt but had forgotten the content. After having written the dream, the participants were asked to estimate the intensity of positive and negative emotions on two four-point scales: 0 = none, 1 = mild, 2 = moderate, and 3 = strong. The participants were asked to write down dream reports on up to five mornings. At the remaining mornings until the fourteenth morning, participants had to mark only if they remembered the content of a dream from the past night or not.

### 2.6. Dream Content Analysis

Dream content was analyzed using scales from Schredl et al. [31] and Schredl and Engelhardt [30]: realism/bizarreness (0 = realistic to 3 = two or more bizarre elements within the dream), positive and negative emotions (two different four-point scales: 0 = none, 1 = mild, 2 = moderate, and 3 = strong), and the number of dream characters. The occurrences of verbal and physical interaction, verbal and physical aggression, characteristics of aggressors, health-related topics, professional environment, leisure, laboratory references, problems, depression, and death related topics were coded binary (1 = present or 0 = not present). Five additional binary scales were constructed for the purpose of this study: reference to and movements of legs and arms, physical activity, and restriction of movements.

The interrater reliability of these scales is satisfactory [32]: realism/bizarreness *r* = 0.765, positive emotions *r* = 0.642, negative emotions *r* = 0.825 (all Spearman rank correlations), for occurrence of verbal interaction judges reached 87% exact agreement.

### 2.7. Procedure

The questionnaires were filled in during the interview and subsequently discussed and corrected if necessary. The RBDQ-HK was implemented at a second interview; the interval was 4.3 ± 1.7 months. The handwritten dream reports were transcribed. Word count was used to determine dream length. The dream reports were rearranged randomly and were rated along the scales described in the dream content analysis section by a, for the diagnoses blind, native Portuguese-speaking judge (J.P.).

### 2.8. Statistical Analysis

SPSS 20.0 (SPSS, Inc., Chicago, IL) and SAS 9.4 (SAS Institute Inc., Cary, NC, USA) were used for statistical analyses. To adjust for multiple dream reports from one participant, mixed models were used according to the variables' measurement levels. We used general linear mixed models for Gaussian data and generalized linear mixed models for ordinal data (ordinal logistic regression with mixed effects). The marginal approach of generalized estimating equations (GEE) was used for analyzing correlated binary data. To compare WD + RBD versus WD − RBD groups, means of emotional scales of each patient and numbers of dreams with aggressive content of each patient were compared in a Mann-Whitney *U* test. Levels of significance were set to alpha 0.05. Being a study of mainly exploratory character, Bonferroni correction was not applied.

## 3. Results

### 3.1. Wilson's Disease Patients Compared to Healthy Controls

Compared to controls, cognitive performance of WD patients measured by the Addenbrooke's Cognitive Examination Revised was significantly lower, especially in the domains of verbal fluency, visuospatial abilities, and memory (Table 1). Also the emotional state in the Beck Depression Inventory and the sleep quality in the Pittsburgh Sleep Quality Index were significantly worse.

Patients with WD showed a significantly reduced dream word count compared to controls. In almost all of the other dream dimensions examined, WD patients showed similar dream characteristics as controls (Tables 2 and 3). DRF, both retrospectively asked and prospectively determined in a two-week diary, and nightmare frequencies were not different between WD patients and controls. In the dream content dimension, only retrospective lifetime questions on violent or aggressive dream content and on frightening and horrifying content (RBDQ-HK4 and 5) were significantly different; they were less often answered positively by WD patients than by controls.

26 of 38 WD patients (68.42%) and 35 of 41 controls (85.37%, n.s.) wrote at least one dream report (Table 3). The total number of dream reports from patients was 112 (last dreams *n* = 21, diary dreams *n* = 91) and from controls 146 (32 and 114, resp.). Dream report contents of WD patients and controls did not differ significantly throughout all the subjectively and judge-rated categories. This also included no difference in frequencies of motor activity themes in dreams of WD patients.

Interviewed before writing the dream reports on whether they already had observed any relationship between WD and dreaming, 15 patients answered affirmatively. Of these 15 patients, only four patients reported WD itself as an explicit theme of a dream. One patient had cried in a dream because of being newly diagnosed with the disease, one had had repeated dreams of losing daily life capacities while starting WD treatment, one had dreamt of never reaching an improvement of the disease, and one had repeatedly dreamt of being able to eat chocolate (which patients have to abstain from because of its copper contents) and of having healthy children without WD. Six patients had had dreams that they could move the body without any disabling condition (being entirely like before the disease; dancing; travelling; speaking and walking normally; speaking and making love as before; going out with friends at night), while two patients had had dreams on motor disability (one dreamt of losing his legs in an accident; another, a teacher, had repeated dreams in the beginning of the disease of having lost her ability to speak and saw herself standing in front of her class having lost her voice).

### 3.2. WD Patients with RBD versus WD Patients without RBD

The implemented questionnaires showed no significant difference between WD patients with and without RBD in cognitive performance and emotional state; sleep quality was considerably worse in WD + RBD, though not reaching significance, probably due to the low number of patients (Table 1).

Compared to WD − RBD, patients with WD + RBD rated consistently a significantly higher nightmare frequency for both the last months and the last year, determined with two different scales at different points in time (Table 2). WD patients with RBD also reported to have significantly more dreams with violent or aggressive content and showed a tendency towards more dreams with frightening and horrifying content during the past year.

The total number of dream reports from four patients with WD + RBD was 18 (last dreams *n* = 3, diary dreams *n* = 15); from 22 patients with WD − RBD it was 94 (18 and 76, resp.; Table 4). Further 12 patients with WD − RBD did not remember dream content in the prospective part of the study. Dream contents of WD patients with RBD showed significantly more self-rated negative emotions, as a tendency also more negative emotions in the judge-rating, and significantly more aggression, in particular more verbal aggression towards the dream ego. The number of reports per patient and the word count per dream report were not different.

### 3.3. Dream Examples from the Diaries

Female patient with WD and RBD, age 20, wrote:

*I was in a school. There were many classrooms. The walls were painted in a certain green, which reminded me both of a hospital, when I was a child, in Monte Verde, and of my elementary school. The school desks were made of wood and I was sitting at a table, looking there at two empty pictures in the background, one black and one white. The bell rings, sounds of rushing, but instead of children, as I expected, appeared my mother, my friend, and many male nurses. They grabbed me and threw me on the floor (my mother cried at the door). I screamed that I wasn't crazy. They injected something into my neck and into the base of my spine. My friend tried to calm me down, but I went on screaming and flailing. They threw me in a car that then right away turned into a room. I cried and didn't see anybody, only myself (from above). Note: I woke up with scratches on my arm.*

Female patient with WD without RBD, age 34, wrote:

*I dreamt that I was at Grandma's house. It was daytime and the weather was very cold. The house was under renovation and I helped painting a wall. It became each time colder and it seemed that the wall was doubling in size. It was as if I had not yet gotten the disease, as if all was still as before. I remember that I felt very good about that.*

Male patient with WD without RBD, age 31, wrote:

*I dreamt that I traveled with my girlfriend to Bahia and we went to the house of my grandmother who lives there. My girlfriend and me were going to the parties there, because we had gone there during the time of Christmas holidays and we danced normally, as if I had no physical problem at all.*

## 4. Discussion

We report first quantitative data on dreaming in WD and in WD with RBD. WD patients showed a significant lower dream word count as compared to age- and sex-matched, healthy controls, but no differences in dream recall. There were very few significant differences between WD patients and healthy controls regarding dream quality and dream content. Interestingly, the only significant group differences in dream characteristics were lifetime occurrences of violent/aggressive and frightening/horrifying dream contents, which WD patients reported significantly less frequently. The subgroup of WD patients with RBD reported significantly more aggressive dream contents, both retrospectively in the questionnaires and prospectively in the diary, and a significantly higher nightmare frequency than WD patients without RBD.

Unexpectedly, we did not find significant changes in DRF in the presence of clear neurological deficits and low ratings in visuospatial abilities, verbal fluency, memory, mood, and sleep quality. Most of our patients were under stable clinical and treatment conditions for already years and most of them had reached an improvement of the initial WD deficits. Considering the existing literature on dreaming under chronic cerebral lesions showing decreases in DRF initially after brain lesions [33], but also that DRF can recover to prelesion levels in the course of time [34], it might be supposed that dreaming had adapted to chronic deficits of the disease. In order to test this hypothesis, it would be necessary to study dream recall during the initial phase of WD and during the following course of successful treatment.

The reduced dream word count in this WD group can be attributed to cognitive deficits in verbal fluency and memory as previous research [35] showed that diary dream length is strongly related to verbal memory. That is, the cognitive deficits in WD affect the process of recalling the dream in full detail. We cannot exclude the possibility that these cognitive deficits might have caused also other influences on dream characteristics of the patients. The data on dream emotions did not confirm our hypothesis as we expected more negatively toned dreams in WD patients due to the burden of their disease, indicated by the higher Beck Depression Inventory scores.

The WD patients reported less lifetime occurrences of violent/aggressive and frightening/horrifying dream contents than controls, a finding that is in line with a previous study [19] showing reduced nightmare frequency in WD patients compared to controls. As these characteristics did not emerge in the prospectively collected diary dreams and the nightmare frequency scale, this finding might reflect a development of dream emotions over the course of the illness.

In our WD sample, movement ratings as well as the other content dimensions investigated were normal. The physical handicap was almost not represented in the dreams. As research indicate that congenital paraplegic patients [12], amputees [36], patients with ataxic disorders and Parkinson's disease [13], and patients with lesional hemiplegia, cortical blindness, and aphasia [16] have dreams with an intact body image, one might speculate that dreams include an innate body image that is not related to waking-life experience. A recent study in amputees [37] indicates that also waking-life experiences like phantom pain can affect the body image in dreams. It would be interesting to study the subjectively perceived impairment in patients with WD and to relate this variable to the percentage of dreams with a physical handicap. The subjective reports of the patients regarding their perceived effect of WD on dreaming indicate such a relation between waking-life and dreaming.

The significant differences in regard to nightmare frequency and aggressive dream contents between the subgroup of WD patients with and without RBD are in accordance with most dream studies in RBD [4–6, 38, 39], even though one study [9] in idiopathic RBD found no heightened aggressiveness in diary dreams. One has to keep in mind, however, that the higher nightmare frequency and more aggressive content might be an artifact, because RBD patients are awaken from these dreams due to their body movements and might recall these dreams more easily; controlled studies with lab awakenings do not show these strong differences [8]. In order to follow up these preliminary findings, it would be interesting to carry out sleep laboratory studies with REM awakenings.

Atypically, all our RBD patients were women. Even though this may be explained by the higher prevalence of women among young RBD patients [40], it is a possible confounder for dream word count and content analysis. In our sample, however, number of dreams and word count did not differ between RBD and non-RBD patients. As women usually have less aggressive dreams than men [6, 41] the finding of aggressive dream content in our female RBD patients is even more meaningful. It is possible that the true frequency of RBD in WD might even be higher than in our cohort, as patients with cognitive deficits may not be aware of RBD symptoms. This possibility gains support by the finding of nine WD patients (4 males, 5 females) in our cohort, who did not fulfill clinical criteria of RBD, but showed increased RWA (two SD above the mean of healthy controls) [15]. To what extent dreaming, on the background of increased RWA, is necessary to trigger off motor behaviour during REM sleep is an open question in the pathophysiology of RBD. In the present ICSD-3 criteria for RBD [3], dream enactment behaviour is not a necessary condition for the diagnosis of RBD. Excessive RWA with repeated, PSG documented motor behaviour during REM sleep fulfill this definition of RBD. Whether such events of RBD are really free of dreaming or if these patients have only no awareness of their dream mentation, for example, due to dementia or other cognitive limits, is difficult to differentiate. On the other hand, RWA does not necessarily produce REM sleep motor behaviour. To better elucidate these connections, it would be of high interest to have solid data on DRF and dream content in various collectives of patients with only RWA, with REM sleep motor behaviour, and with fully developed RBD. This might shed more light on the significance of dreaming in the development of RBD. Another study on WD patients, implementing an RBD sleep questionnaire, found “clinical symptoms suggesting the possibility of RBD” in even 47.3% and “vivid dreams” in 23.6% of these patients [18]. However, the low number of WD patients with RBD and the low number of reported dreams available, as well as possible medication influences, are clear limits of the present study, so that the findings should be cautiously interpreted.

To summarize, the first study systematically investigating dreaming in WD showed that RBD in WD had a clear influence on dreaming and that the influence of WD on dreaming was minor. Nevertheless, the finding of reduced dream length indicated that cognitive deficits in WD have an effect on dream recall. Further studies, especially longitudinal observations from early diagnosis to successful treatment, should be carried out. In order to shed more light on the effect of waking-life symptoms on dream content, it would be advisable to elicit the subjectively experienced burden of the disease in these patients by using questionnaires or diaries and to relate this to dream content. The interesting findings in our small group of WD patients with RBD should be complemented with larger samples. Overall, the findings indicate that dreaming is affected by a severe neurological disease like Wilson's disease and that it is important to follow these patients in longitudinal designs.

## Figures and Tables

**Table 1:** Cognitive, affective, and sleep characteristics in patients with Wilson's disease versus healthy controls and in WD with RBD versus WD without RBD (mean ± SD).

	Wilson's disease (*n* = 38)	Wilson's disease without RBD (*n* = 34)	Wilson's disease with RBD (*n* = 4)	Healthy controls (*n* = 41)	All WD versus HC *p*	WD + RBD versus WD − RBD *p*
Female/male	17/21	13/21	4/0	17/24	ns	0.019
Age [yrs]	34.84 ± 9.42	35.35 ± 9.48	30.50 ± 8.81	34.32 ± 10.18	ns	ns
Severity of Wilson's disease, UWDRS [0–320]	68.82 ± 41.12	69.91 ± 42.11	59.50 ± 34.84		—	ns
Addenbrooke's Cognitive Examination [0–100]	84.00 ± 10.53	83.53 ± 10.99	88.00 ± 3.83	91.12 ± 5.52	<0.001	ns
ACE-R1: attention/orientation [0–18]	16.74 ± 1.75	16.71 ± 1.84	17.00 ± 0.82	17.20 ± 1.21	ns	ns
ACE-R2: memory [0–26]	20.90 ± 3.85	20.74 ± 3.96	22.25 ± 2.75	22.61 ± 2.93	0.028	ns
ACE-R3: verbal fluency [0–14]	8.68 ± 3.31	8.41 ± 3.33	11.00 ± 2.16	11.37 ± 1.89	<0.001	ns
ACE-R4: language [0–26]	24.63 ± 1.82	24.53 ± 1.90	25.50 ± 0.58	24.93 ± 1.86	ns	ns
ACE-R5: visuospatial abilities [0–16]	13.05 ± 2.92	13.15 ± 2.95	12.25 ± 2.99	15.02 ± 1.24	<0.001	ns
Beck Depression Inventory [0–63]	10.66 ± 9.71	10.09 ± 9.55	15.50 ± 11.21	5.44 ± 4.28	0.004	ns
Pittsburgh Sleep Quality Index [0–21]	6.76 ± 4.55	6.47 ± 4.61	9.25 ± 3.59	4.61 ± 2.57	0.013	ns

ACE-R1–5: Addenbrooke's Cognitive Examination subscore from 1 to 5; HC: healthy controls; RBD: REM sleep behaviour disorder; UWDRS: Unified Wilson's Disease Rating Scale; WD: Wilson's disease; WD + RBD: Wilson's disease with REM sleep behaviour disorder; WD – RBD: Wilson's disease without REM sleep behaviour disorder.

**Table 2:** Dream and nightmare frequencies and retrospective dream characteristics (mean ± SD and percentages).

	Wilson's disease (*n* = 38)	Wilson's disease without RBD (*n* = 34)	Wilson's disease with RBD (*n* = 4)	Healthy controls (*n* = 41)	All WD versus HC *p*	WD + RBD versus WD − RBD *p*
Dream recall frequency during last months [*n*/week]	1.84 ± 2.30	1.71 ± 2.27	3.00 ± 2.61	1.95 ± 2.06	ns	ns
Nightmare recall frequency during last months [*n*/month]	1.18 ± 2.33	0.86 ± 1.54	3.94 ± 5.46	0.65 ± 1.03	ns	0.040
Dream recall frequency, two-week diary [*n*/week]	1.32 ± 1.45	1.27 ± 1.48	1.75 ± 1.19	1.48 ± 1.41	ns	ns
RBD Questionnaire-Hong Kong, total score [0–100]	15.54 ± 12.13	12.79 ± 9.01	38.25 ± 11.35	13.71 ± 5.67	ns	<0.001
*RBDQ-HK1: Did you often have dreams?*						
Lifetime occurrence [y/n]	78.38%	75.76%	100.00%	92.68%	ns	ns
Recent 1-year frequency [0–4]	2.32 ± 1.44	2.18 ± 1.45	3.50 ± 0.58	2.78 ± 1.11	ns	ns
*RBDQ-HK2: Did you often have nightmares?*						
Lifetime occurrence [y/n]	59.46%	54.55%	100.00%	68.29%	ns	ns
Recent 1-year frequency [0–4]	0.73 ± 0.99	0.49 ± 0.71	2.75 ± 0.50	0.73 ± 0.81	ns	0.001
*RBDQ-HK3: Did you have dreams with an emotional and sorrowful content?*						
Lifetime occurrence [y/n]	70.27%	66.67%	100.00%	75.61%	ns	ns
Recent 1-year frequency [0–4]	0.92 ± 1.04	0.82 ± 0.92	1.75 ± 1.71	1.00 ± 0.84	ns	ns
*RBDQ-HK4: Did you have dreams with a violent or aggressive content (e.g., fighting with someone)?*						
Lifetime occurrence [y/n]	43.24%	39.39%	75.00%	68.29%	0.026	ns
Recent 1-year frequency [0–4]	0.43 ± 0.80	0.33 ± 0.69	1.25 ± 1.26	0.51 ± 0.64	ns	0.028
*RBDQ-HK5: Did you have dreams with a frightening and horrifying content (e.g., being chased by a ghost)?*						
Lifetime occurrence [y/n]	37.84%	33.33%	75.00%	65.85%	0.013	ns
Recent 1-year frequency [0–4]	0.43 ± 0.77	0.30 ± 0.59	1.50 ± 1.29	0.29 ± 0.46	ns	0.051

HC: healthy controls; RBD: REM sleep behaviour disorder; RBD-HK1–5: RBD Questionnaire-Hong Kong questions 1 to 5; WD: Wilson's disease; WD + RBD: Wilson's disease with REM sleep behaviour disorder; WD − RBD: Wilson's disease without REM sleep behaviour disorder.

**Table 3:** Self-rated dream emotions and dream content analysis by external judge; Wilson's disease versus healthy controls (mean ± SD and percentages).

	Wilson's disease (*n* = 26)	Healthy controls (*n* = 35)	All WD versus HC *p*
Female/male	12/14	15/20	ns
Dream reports per participant [*n*]	4.31 ± 2.06	4.17 ± 2.26	ns
Word count per dream report [*n*]	74.52 ± 69.27	98.30 ± 61.41	0.006
Bizarreness [0–3]	0.65 ± 0.98	0.91 ± 1.10	ns^1^
Positive emotions (self-rating) [0–3]	1.31 ± 1.26	1.48 ± 1.09	ns^1^
Negative emotions (self-rating) [0–3]	1.21 ± 1.27	1.31 ± 1.06	ns^1^
Positive emotions (external judge) [0–3]	0.49 ± 0.89	0.51 ± 0.90	—^2^
Negative emotions (external judge) [0–3]	0.73 ± 1.07	0.70 ± 1.03	ns^1^
Number of characters [*n*]	1.55 ± 1.44	2.03 ± 1.60	ns^1^
Number of male persons [*n*]	0.59 ± 0.89	0.81 ± 1.01	ns^1^
Number of female persons [*n*]	0.71 ± 0.82	0.64 ± 0.86	ns^1^
Verbal interaction [y/n]	49.11%	52.74%	ns^1^
Physical interaction [y/n]	14.29%	21.92%	ns^1^
Health-related topics [y/n]	5.36%	2.74%	ns^1^
Professional environment [y/n]	7.14%	10.96%	ns^1^
Leisure [y/n]	48.21%	54.79%	ns^1^
Problems [0–2]	0.41 ± 0.64	0.53 ± 0.68	ns^1^
Movements of legs [y/n]	25.00%	35.62%	ns^1^
Movements of arms [y/n]	18.75%	26.71%	ns^1^
Reference to legs or arms [y/n]	5.36%	2.74%	ns^1^
Physical activity [y/n]	15.18%	14.38%	ns^1^
Restriction of movements [y/n]	1.79%	3.42%	ns^1^
Verbal and physical aggression towards dream ego [y/n]	9.82%	8.22%	ns^1^
Aggression, total [y/n]	15.18%	10.96%	ns^1^

*Notes*. ^1^Group effect, word count was included as covariate. ^2^Algorithm did not converge.

HC: healthy controls; WD: Wilson's disease.

**Table 4:** Dream content analysis of aggressive themes; WD with RBD versus WD without RBD (mean ± SD and percentages).

	Wilson's disease with RBD (*n* = 4)	Wilson's disease without RBD (*n* = 22)	WD + RBD versus WD − RBD *p*	Effect size
Female/male	4/0	8/14	0.19	0.976
Dream reports per patient [*n*]	4.50 ± 2.65	4.27 ± 2.00	ns	0.098
Word count per dream report [*n*]	69.90 ± 31.47	66.83 ± 43.87	ns	0.080
Positive emotions (self-rating) [0–3]	0.98 ± 0.90	1.51 ± 0.96	ns	−0.570
Negative emotions (self-rating) [0–3]	2.58 ± 0.52	1.03 ± 1.02	0.031	1.915
Positive emotions (external judge) [0–3]	0.48 ± 0.36	0.53 ± 0.70	ns	−0.090
Negative emotions (external judge) [0–3]	1.25 ± 0.64	0.58 ± 0.63	0.081	1.055
Dreams with verbal aggression towards others [*n*]	0.50 ± 0.58	0.27 ± 0.55	ns	0.406
Dreams with verbal aggression towards dream ego [*n*]	1.00 ± 0.82	0.05 ± 0.21	0.021	1.587
Dreams with physical aggression towards others [*n*]	0	0.05 ± 0.21	ns	−0.337
Dreams with physical aggression towards dream ego [*n*]	0.75 ± 1.50	0.18 ± 0.40	ns	0.519
Dreams with verbal and physical aggression towards dream ego [*n*]	1.50 ± 1.29	0.23 ± 0.43	0.048	1.321
Dreams with aggression, total [*n*]	1.75 ± 0.96	0.46 ± 0.67	0.016	1.558

WD + RBD: Wilson's disease with REM sleep behaviour disorder; WD − RBD: Wilson's disease without REM sleep behaviour disorder.
